# Effects of maternal depression and prenatal SSRI exposure on executive functions and susceptibility to household chaos in 6-year-old children: prospective cohort study

**DOI:** 10.1192/bjo.2020.73

**Published:** 2020-09-07

**Authors:** Gurpreet Dhaliwal, Whitney M. Weikum, Alexia Jolicoeur-Martineau, Ursula Brain, Ruth E. Grunau, Tim F. Oberlander

**Affiliations:** Division of Developmental Pediatrics, University of British Columbia, Vancouver, Canada; Division of Developmental Pediatrics, University of British Columbia, Vancouver, Canada; Jewish General Hospital, Montreal, Canada; BC Children's Hospital Research Institute, Vancouver, Canada; University of British Columbia, Vancouver; and BC Children's Hospital Research Institute, Vancouver, Canada; University of British Columbia, Vancouver; and BC Children's Hospital Research Institute, Vancouver, Canada

**Keywords:** Antidepressants, executive functions, household chaos, prenatal mood disorders, perinatal psychiatry

## Abstract

**Background:**

Maternal depressed mood during pregnancy may shape a child's adaptation to their environment and engagement in goal-directed behaviour such as executive functions. Whether everyday household context also alters executive functions in children with prenatal selective serotonin reuptake inhibitor (SSRI) antidepressant exposure remains to be determined.

**Aims:**

To examine the impact of prenatal depressed maternal mood and SSRI exposure on child executive functions and to determine whether these exposures shape a susceptibility to household chaos.

**Method:**

A prospective cohort study of mothers and their children (118 mother–children dyads (47 SSRI-exposed, 71 non-exposed)) followed from the second trimester to 6 years. Regression models examined relationships between maternal depressed mood and household chaos on maternal report of child executive functions. Competitive-confirmatory regression models examined whether children were susceptible to household chaos or were positively influenced by less chaos.

**Results:**

Prenatal SSRI exposure, third-trimester maternal depressed mood and household chaos in a three-way interaction were associated with executive functions within a model of differential susceptibility. When household chaos was low, children of non-prenatally depressed mothers had better executive function than children of prenatally depressed mothers, regardless of whether the mothers were SSRI-treated. However, when household chaos was high, SSRI-exposed children of mothers who were not depressed during pregnancy had poorer executive functions at 6 years of age compared with SSRI-exposed children whose mothers were symptomatic during pregnancy.

**Conclusions:**

The impact of household chaos depended on whether mothers were prenatally depressed and whether mothers were SSRI-treated.

Maternal mental health and its impact on childhood cognitive development and behaviour^1^ have been widely reported, but studies continue to yield small to moderate effect sizes.^2^ Developmental outcomes vary and it is often unclear why some, but not all, children are negatively affected, or even why some children benefit under positive environmental conditions.

Long before birth, maternal mental health can shape diverse biological and behavioural processes that regulate arousal, attention and thinking in ways that facilitate goal-directed behaviour.^3^ These higher-order cognitive tasks, termed executive functions,^4^ include cognitive flexibility, inhibition and working memory. These constructs reflect ability to plan, organise and focus attention^1^ and affect childhood behaviour and academic achievement.^5^

## Prenatal exposure to SSRIs, and household chaos

Although executive functions are heritable, they are susceptible to early-life environmental factors such as chronic exposure to depressed maternal mood during pregnancy. Inherent to maternal mental health during pregnancy are the selective serotonin reuptake inhibitor (SSRI) antidepressants widely used to manage prenatal maternal mood disturbances, adding another early-life exposure that potentially shapes brain development and emerging executive functions.

Executive functions are dependent on multiple neurotransmitters, including serotonin (5-hydroxytryptamine, 5-HT), the very target of SSRIs. SSRIs primarily act by blocking reuptake of serotonin transporter protein (5-HTT), increasing the extent to which intrasynaptic serotonin remains active and available for neurotransmission. SSRIs readily cross the blood–brain barrier and placenta,^6^ therefore it is conceivable that early changes in 5-HT signalling have a developmental impact on systems that subsequently regulate self-control and executive functions.^7^ Changes in serotonin signalling in prefrontal cortical networks have been linked with cognition, although findings are inconsistent.^8^ This raises intriguing questions about what might underlie developmental variations.

Disorganised, noisy and unpredictable environments affect the development of executive functions^9^ and may lead to increased conduct problems and lower IQ in kindergarten-age children.^10^ Further, household chaos is highly intertwined with parental depression and both have been associated with poorer child executive functions.^1^ Yet, even in disorganised and busy circumstances, some children remain resilient and understanding why some children are susceptible to adversity and others are not remains a pressing question.

## ‘Differential susceptibility’ and ‘vantage sensitivity’

To date research has focused on identifying adverse developmental outcomes in children with prenatal SSRI exposure, such as attention-deficit hyperactivity disorder (ADHD), autism spectrum disorder and anxiety.^11^ This has led to a focus on ‘diathesis-stress’ models to explain why some children may be more negatively affected by adverse environmental conditions.^11^ However, the developmental impact of prenatal SSRI exposure remains inconsistent and contradictory.^6^ Beyond a direct effect related to prenatal exposure to SSRIs or depressed maternal mood, interactions with daily life may shape variations in child development. To further understand the impact of interactions between the environment (i.e. household chaos) and biological vulnerabilities (i.e. prenatal maternal depression, prenatal SSRI exposure), the concept of ‘differential susceptibility’ has been proposed to explain why some children may have better outcome than other children under positive environmental conditions and worse outcome under adverse environmental conditions.^12,13^ An alternative theory is ‘vantage sensitivity’, which suggests that some children may have better outcomes under positive environmental conditions than other children^14,15^ (see Jolicoeur-Martineau *et al* ^15^ for a review of the concepts of ‘differential susceptibility’ and ‘vantage sensitivity’).

## The hypothesis

**Fig. 1:**
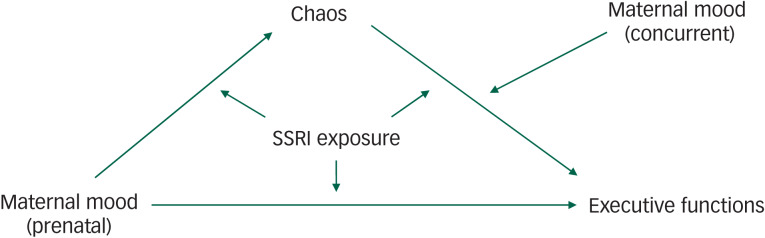
Graphic representations of the competitive-confirmatory model and relationships between the different elements affecting child executive functions at 6 years of age.

To understand variations in behaviour in children with prenatal SSRI exposure, we investigated the impact of both prenatal exposure to SSRIs and depressed maternal mood on early executive functions at 6 years of age, and whether the impact of these exposures shapes a response to household chaos (Fig. 1). We expected that prenatal exposure to SSRIs and maternal depressed mood in the context of increased household chaos might be associated with poorer executive functions. However, given that prenatal SSRI exposure has been associated with improved or sustained executive functions in animal models^8^ and humans,^7^ prenatal SSRI exposure might ‘buffer’ a child from the effects of prenatal maternal mood and thereby shapes a differential susceptibility to a stressful everyday household context. In this sense, using competitive-confirmatory analyses^15^ we expected that prenatal depressed maternal mood and SSRI exposure would interact to reflect differential susceptibility, or even a vantage sensitivity to household chaos.

## Method

Approval was obtained from the University of British Columbia Clinical Research Ethics Board and the Children's and Women's Health Centre of British Columbia Clinical Research Ethics Board (H00-70500, H05-70629, H08-01712). Written informed consent was obtained from all mothers. All procedures contributing to this work comply with the ethical standards of the relevant national and institutional committees on human experimentation and with the Helsinki Declaration of 1975, as revised in 2008.

Mothers (*n* = 191) were recruited during their second trimester of pregnancy either through self-referrals from the community or through referrals from the departments of reproductive psychiatry and family medicine at BC Women's Hospital, Vancouver, Canada, between 2003 and 2009. Mothers and children were studied again when the children had reached 6 years of age, between 2009 and 2014. Inclusion criteria were singleton pregnancy, gestational age at birth (>37 weeks) and no fetal anomalies on antenatal ultrasounds. Exclusion criteria were presence of maternal bipolar disorder, concurrent illicit drug use and maternal medical conditions (e.g. diabetes, hypertension). Of the initial recruits, 8 withdrew before delivery, another 15 withdrew before the end of the child's first year and, by 6 years, 50 declined to participate, were unavailable for study (families had moved, etc.) or there were significant incomplete study data. This left 118 mother–child dyads (47 prenatally SSRI-exposed and 71 non-SSRI-exposed) available for this study. Comparison of the mothers originally recruited and their children who did not participate (*n* = 115 (60.21%) not SSRI-exposed, *n* = 76 (39.79%) SSRI-exposed) showed demographic similarities on birthweight, birth length, head circumference, gender, Apgar score and maternal education. However, in the non-participating group, maternal age was younger (32 *v*. 34 years, *P* = 0.03), prenatal depressed mood was slightly higher (mean Hamilton Rating Scale for Depression (HRSD) score 9.4 *v*. 7.3, *P* = 0.02) and gestational age was shorter (39.1 *v*. 39.7 weeks, *P* = 0.04).

### Maternal mood

Maternal depressed mood was assessed at 26 weeks, 36 weeks and 6-year follow-up using the clinician-rated Hamilton Rating Scale for Depression (HRSD),^16^ yielding a range of symptoms reflecting no depression (0–7), mild depression (8–16), moderate depression (17–23) and severe depression (>23). Prenatal mood (HRSD score at 26 and 36 weeks) was tabulated as a mean of prenatal depressed mood. Initially, maternal mood was used as a continuous measure in the regression models. Then to investigate interactions between maternal mood and SSRI exposure, we used categorical approaches to examine how maternal mood was associated with offspring executive functions. An HRSD score >8 was used to denote the presence of mild to moderate depressive symptoms and to categorically separate depressed from non-depressed mothers.^17^ In addition, the self-report Beck Depression Index (BDI) was obtained at 3-year follow-up because data were obtained at 3 years using a mailing of questionnaires.

### Home environment

Household chaos offers a measure of an environment that is high in noise and crowding and low in regularity^9^ and has been linked to poorer child behaviour.^10^ Home environment was assessed using the Confusion, Hubbub and Order Scale (CHAOS) at age 6. This is a self-report scale (completed here by the mothers) developed as a 15-item measure of home chaos and disorganisation.^9^ Higher scores on this scale are indicative of more disorganised, confusing and noisy homes.

### Executive functions

Children's behaviour was assessed using the Behavior Rating Inventory of Executive Function (BRIEF) – an 86-item parent-completed rating scale widely used in clinical practice.^18^ The BRIEF's Global Executive Composite Index reflects the behavioural manifestation of executive function (i.e. inhibition, shift, emotional control, initiation, planning). The BRIEF comprises eight scales (Working memory, Inhibit, Shift, Emotional control, Initiate, Plan/Organise, Organisation of Materials, Monitor), which yields two Indexes: (a) Metacognition, reflecting the ability to cognitively self-manage tasks and monitor performance; and (b) Behavioural Regulation, the ability to shift and modulate emotions and behaviour via appropriate inhibitory control. In this study, the BRIEF's Global Executive Composite score was used as the key dependent measure of overall executive functions. Higher scores indicate poorer executive functions.

### Statistical analysis

Regression modelling was utilised to examine relationships between SSRI exposure, CHAOS score, pre- and postnatal maternal depression (HRSD) scores, and child BRIEF Global Executive Composite *T*-scores. Measures of postnatal maternal mood (when the infant was 6 months and 3 years) were also obtained but, owing to high collinearity between measures, these were not used in our analytic models. Postnatal maternal mood at 6 years postpartum was included in our models as a potential confounder, as maternal depressed mood may influence maternal perception of household chaos and child executive functions.

Models were constructed in a stepwise manner: step 1 was for main effects only, step 2 for main effects and two-way interaction and step 3 for main effects, two-way interaction and three-way interaction. Initially, models used prenatal maternal depression as a continuous variable, then, to examine additive interactions, a dichotomous measure of prenatal maternal depression was used (coded as 1 when HRSD ≥ 8 and 0 otherwise). We used the following reparametrised equations:
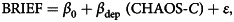
for children exposed to perinatal depression (with or without SSRI exposure);
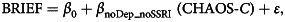
for children non-exposed to both perinatal depression and SSRIs; and
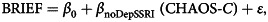
for children non-exposed to perinatal depression but exposed to SSRIs.

The parameters were the intercept (*β*_0_), the slope for children exposed to perinatal depression (*β*_dep_), the slope for children non-exposed to both perinatal depression and SSRIs (*β*_noDep–noSSRI_), the slope for children non-exposed to perinatal depression but exposed to SSRIs (*β*_noDep–SSRI_) and the cross-over point between the two slopes (*C*). The cross-over point (*C*) distinguishes a vantage sensitivity from differential susceptibility. Namely, if *C* ≠ max(CHAOS) (differential susceptibility), then the two lines cross over in the middle of the graph and the children non-exposed to prenatal depression may have a worse outcome than the exposed children when CHAOS > *C*. Alternatively, if *C* = max(CHAOS) (vantage sensitivity), then the two lines meet at the right of the graph (no cross-over) and children non-exposed to depression cannot have a worse outcome than children exposed to depression, at any level of household chaos. Both the differential susceptibility and vantage sensitivity models assume that children exposed to perinatal depression would not be influenced by the environment (i.e. that *β*_dep_ = 0). However, as there remains a possibility that the environment exerts a slight effect even on exposed children, the vantage sensitivity and differential susceptibility models are further separated into two groups: a weak model (*β*_dep_ ≠ 0, *β_dep_* < *β*_noDep_noSSRI_ and *β*_dep_ < *β*_noDep_noSSRI_) and a strong model (*β*_dep_ = 0).

In strong models, some individuals are affected by the environmental exposure of interest, whereas others are not. In weak models, all are affected by the environmental exposure, but some more strongly than others.^15^ We measured the effect size using the coefficient of determination (*R*^2^).

### Competitive-confirmatory analysis

To test whether the interaction between mother's perinatal depressed mood and household CHAOS reflected diathesis–stress, differential susceptibility or vantage sensitivity, we used competitive-confirmatory analyses.^15^ The Akaike information criteria (AIC) and the 95% confidence interval of the cross-over point were used to determine which of the four models best fit the data. Only the vantage sensitivity and strong differential susceptibility model testing are reported; all models were tested using Statistical Package for the Social Sciences (SPSS) for Windows^19^ and SAS System 9.4 for Windows.^20^

## Results

Maternal characteristics were generally similar for both SSRI-exposed and non-exposed groups (Table 1) and for subgroups of non-SSRI-treated mothers (depressed versus non-depressed (Table 2); however, mothers treated with an SSRI had higher depression symptom scores (HRSD) and fewer years of education (a proxy measure of socioeconomic status) than mothers not taking SSRIs. For years of education, in the SSRI-treated mothers the mean was 16.23 years (s.d. = 3.39) and in the non-SSRI-treated mothers it was 17.87 years (s.d. = 3.03), reflecting a slightly higher middle-class status. Mothers reported a mean 240 days (s.d. = 68) of SSRI treatment during their pregnancy. There were no significant differences between the cohorts in levels of home environment (CHAOS score) (Table 1). Maternal report of home environment was associated with depressed mood at 6 years (*R*^2^ = 0.357; *P* < 0.01) but not prenatal depression symptoms (*R*^2^ = 0.146; *P* = 0.113). We found no significant differences in BRIEF scores between SSRI-exposed and non-exposed groups. However, prenatally depressed mothers (HRSD ≥ 8) had children with significantly poorer BRIEF scores (50.76 *v*. 56.63; *P* < 0.001) and reported significantly higher CHAOS scores (20.63 *v*. 18.97; *P* = 0.004); neither gender, education nor mother's age at childbirth were significant in the model. None of the children were being treated with a psychotropic medication at the time of the 6-year follow-up.

**Table 1:** Mother–child cohort characteristics

	SSRI-exposed (*n* = 47)	Non-SSRI-exposed (*n* = 71)	*P*
Maternal characteristics			
Age at childbirth, years: mean (s.d.)	33.06 (5.67)	34.38 (5.15)	0.096
Education, years: mean (s.d.)	16.32 (3.36)	17.89 (3.01)	<0.05*
Prenatal depression symptoms, HRSD score: mean (s.d.)	10.32 (5.06)	5.27 (5.01)	<0.05*
Prenatal SSRI use, days: mean (s.d.)	240 (68.0)	N/A	
Prenatal SSRI type, *n*			
Paroxetine	16		
Fluoxetine	6		
Sertraline	5		
Venlafaxine	13		
Citalopram	7		
Prenatal SSRI median dose, mg/day			
Paroxetine	27.5		
Fluoxetine	35		
Sertraline	50		
Venlafaxine	150		
Citalopram	30		
Mood 6 months postpartum, HRSD score: mean (s.d.)	9.00 (6.56)	5.20 (5.39)	<0.05*
Mood 3 years postpartum, BDI score: mean (s.d.)	10.9 (9.10)	6.23 (7.30)	<0.05*
Mood 6 years postpartum, HRSD score: mean (s.d.)	10.8 (6.6)	6.32 (5.7)	<0.05*
Prenatal alcohol intake, drinks: mean (s.d.)^a^	3.64 (6.3)	3.36 (6.04)^b^	0.71
Prenatal smoking frequency (<20 cigarettes/day)	1	1^b^	0.74
Child characteristics			
Gender, m:f	16:31	37:34	0.054
Gestational age, weeks: mean (s.d.)	39.27 (1.56)	39.95 (1.41)	0.02*
Birthweight, g	3245.20 (499.49)	3517.31 (457.13)	<0.05*
Apgar score at 1 min: mean (s.d.)	7.30 (1.77)	8.39 (1.27)	<0.05*
Apgar score at 5 min: mean (s.d.)	8.77 (0.73)	9.08 (0.44)	<0.05*
BRIEF GEC score at 6 years: mean (s.d.)	53.91 (9.68)	53.97 (8.69)	0.58
Age at 6-year follow-up, years: mean (s.d.)	6.05 (0.75)	5.86 (0.59)	0.13
Environmental characteristics			
CHAOS score sum, mean (s.d.)	20.19 (3.24)	19.38 (3.10)	0.18

SSRI, selective serotonin reuptake inhibitor; HRSD, Hamilton Rating Scale for Depression; BDI, Beck Depression Inventory; BRIEF, Behavior Rating Inventory of Executive Function; GEC, Global Executive Composite; CHAOS, Confusion, Hubbub and Order Scale.

a. Alcohol intake was measured by the number of drinks (units of alcohol) across pregnancy.

b. *n* = 69.

* *P* < 0.05.

**Table 2:** Maternal characteristics of subgroup of mothers not treated with an antidepressant during pregnancy

	Not prenatally depressed (*n* = 58)	Prenatally depressed (*n* = 13)	*P*
Maternal characteristics			
Age at childbirth, years: mean (s.d.)	34.0 (4.5)	36.3 (7.4)	0.137
Education, years: mean (s.d.)	18.0 (2.9)	17.5 (3.6)	0.578*
Prenatal depression symptoms, HRSD score: mean (s.d.)	3.3 (2.4)	14.1 (4.4)	<0.05*
Mood 6 months postpartum, HRSD score: mean (s.d.)	3.9 (4.2)	11.25 (6.4)	<0.05*
Mood 3 years postpartum, BDI score: mean (s.d.)	4.1 (4.2)	19.2 (8.4)	<0.05*
Mood 6 years postpartum, HRSD score: mean (s.d.)	5.0 (4.8)	12.1 (6.0)	<0.05*
Prenatal alcohol intake, drinks: mean (s.d.)^a^^,^^b^	3.9 (6.3)	2.2 (4.4)	0.85
Prenatal smoking frequency (<20 cigarettes/week)^b^	1	0	0.77

HRSD, Hamilton Rating Scale for Depression; BDI, Beck Depression Inventory.

a. Alcohol intake was measured by the number of drinks across pregnancy.

b. *n* = 69.

* *P* < 0.05.

### Primary analysis: using continuous prenatal depression

Regression modelling examining the influence of prenatal maternal mood, SSRI exposure and household chaos on BRIEF scores, controlling for concurrent maternal mood (Table 3), demonstrated main effects for prenatal mood, SSRI exposure and CHAOS score. A two-way interaction emerged between SSRI exposure and prenatal maternal mood, suggesting that for mothers who took antidepressants prenatally, the more depressed they were prenatally (i.e. they did not benefit from pharmacotherapy), the less self-regulated (higher BRIEF scores) their child appeared to be at age 6. Additionally, when CHAOS scores were added, a three-way interaction emerged between prenatal mood, SSRI exposure and household chaos, suggesting that for mothers who took antidepressants prenatally, the more depressed they were prenatally, the less susceptible their children were to a chaotic household environment. Postnatal maternal depression was not significantly associated with BRIEF scores.

**Table 3:** Prediction of children's BRIEF scores at 6 years of age from the interaction of prenatal maternal depression (continuous or binary), SSRI exposure and CHAOS score (*n* = 118)^a^^,^^b^

Predictors	Continuous depression Step 1	Continuous depression Step 2	Continuous depression Step 3	Binary depression Step 1	Binary depression Step 2	Binary depression Step 3
Intercept	35.95***	35.70***	26.67***	38.56***	38.51***	26.11***
SSRI exposure	−1.98	−6.90*	−6.78*	−2.58	−3.33	−3.87
Prenatal maternal depression	0.50**	0.27	0.20	6.05**	5.29^t^	4.13
CHAOS score	0.76**	0.84**	1.32***	0.69*	0.70*	1.35***
SSRI exposure × prenatal maternal depression		0.59^t^	2.69**		1.61	43.18***
SSRI exposure × prenatal maternal depression × CHAOS score			−0.11**			−2***
Covariate: maternal depression at 6-year follow-up	−0.05	−0.06	−0.05	−0.02	−0.02	−0.03
*R*^2^	0.15	0.18	0.23	0.16	0.16	0.26
AIC (smaller is better)	510.04	508.53	503.14	509.19	511	497.76

BRIEF, Behavior Rating Inventory of Executive Function; SSRI, selective serotonin reuptake inhibitor; CHAOS, Confusion, Hubbub and Order Scale; AIC, Akaike information criteria.

a. Maternal prenatal and postnatal depression were assessed using the Hamilton Rating Scale for Depression (HRSD). Prenatal depression as binary is defined as 1 when HRSD ≥ 8, and 0 otherwise.

b. Main effects: prenatal mood, β = 0.20, 95% CI −0.23 to 0.62; SSRI exposure, β = −6.78, 95% CI −12.98 to −0.59; and CHAOS scores, β = 1.32, 95% CI 0.69 to 1.95. Two-way interaction: SSRI exposure and prenatal maternal mood, β = 2.70, 95% CI 1.02 to 4.38. Three-way interaction: prenatal mood, SSRI exposure and CHAOS scores, β = −0.11, 95% CI −0.18 to −0.03. Postnatal maternal depression was not associated with BRIEF scores (*P* > 0.05).

*^t^P* < 0.10, *P < 0.05, **P < 0.01, ***P < 0.001.

### Secondary analysis: dichotomised prenatal depression

To further examine these interactions and investigate the role of prenatal maternal depressed mood in particular, a dichotomised prenatal maternal mood variable (coded 1 when HRSD ≥ 8, and 0 otherwise) was used to distinguish mild to moderate depressive symptoms from low non-impairing symptoms in a regression model. In this model the influence of prenatal maternal mood, prenatal SSRI exposure and household chaos on child BRIEF scores was examined, accounting for postnatal maternal mood (as a continuous variable) (Table 3). Main effects were observed for CHAOS score (β = 1.35, 95% CI 0.76 to 1.95), but were not observed for prenatal mood (β = 4.13, 95% CI −0.96 to 9.22) or prenatal SSRI exposure (β = −3.87, 95% CI −8.63 to 0.89). Maternal depressed mood at 6 years was not associated with BRIEF scores (β = −0.03, 95% CI −0.31 to 0.26).

A two-way interaction was also apparent between SSRI exposure and prenatal maternal mood, which suggests that mothers who took SSRI medication and were prenatally depressed had children with poorer executive functions (higher BRIEF scores) (β = 43.18, 95% CI 21 to 65.36).

In addition, a three-way interaction between prenatal mood, SSRI exposure and CHAOS scores also emerged, suggesting that children's prenatal exposure to antidepressants and maternal depression was associated with less susceptibility to household chaos (β = −2, 95% CI −3.01 to −0.98). This model had a considerably lower AIC (497.76) than the model with continuous prenatal maternal depression (503.14), suggesting that prenatal maternal depression may exert an effect when it is observed clinically but not as depression increased, thus acting in a binary fashion rather than continuously.

In this model, executive functions among children whose mothers were at least mildly symptomatically depressed during pregnancy (i.e. HRSD > 8) remained relatively stable even with increasing levels of household CHAOS scores, regardless of their prenatal SSRI exposure status: SSRI-exposed, β = −0.67, 95% CI −1.53 to 0.18, *P* = 0.12; non-exposed, β = 1.01, 95% CI −0.53 to 2.55, *P* = 0.20. However, for children of non-depressed mothers (i.e. HRSD < 8), increased household chaos was associated with poorer executive functions regardless of SSRI-exposed status (SSRI-exposed, β = 1.54, 95% CI 0.16 to 2.93, *P* = 0.03; non-exposed, β = 1.34, 95% CI 0.69 to 1.99, *P* = 0.0001).

### Competitive-confirmatory analyses

**Fig. 2:**
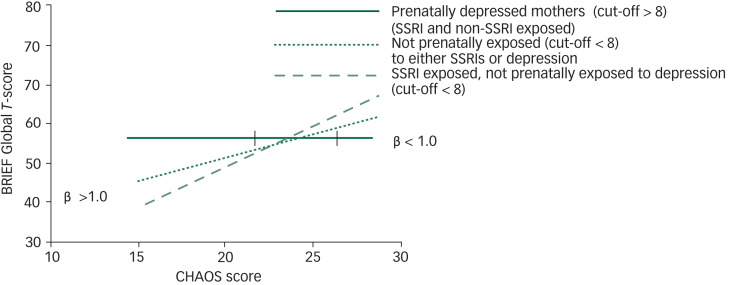
Confirmatory model with the best fit (differential susceptibility strong model) (refer to Table 4).

Competitive-confirmatory analyses were used to determine whether the interactions reflected a differential susceptibility or a vantage sensitivity. For these analyses an *n* = 139 was used, given that postnatal depression measures did not contribute to self-regulation at 6 years. For children exposed to prenatal maternal depression (SSRI-exposed or not), CHAOS scores were not significantly associated with BRIEF scores, thus we considered these children to be non-susceptible (solid green line, Fig. 2). We also tested whether those exposed to both SSRI and maternal prenatal depression would constitute a non-susceptible group, given a negative slope that approached zero (β = −0.67). The model with this grouping had a poorer fit (based on the AIC) and was not considered further. The best model was the differential susceptibility strong model (AIC = 592.94), and in order from best to worst, the other models were vantage sensitivity strong (AIC = 593.79), differential susceptibility weak (AIC = 594.74) and vantage sensitivity weak (AIC = 595.11) (Table 4).

Relationships include the impact of the multiple effects of selective serotonin reuptake inhibitor (SSRI) treatment and exposure (i.e. improved prenatal and postnatal maternal mood, child brain development), maternal mood (pre- and postnatal) and the role of household chaos. We used this model to test the hypothesis that prenatal depressed maternal mood and SSRI exposure would interact to reflect a differential susceptibility, or even a vantage sensitivity, to household chaos.

**Table 4:** Confirmatory models (*n* = 139)^a^^,^^b^

Predictors	Differential susceptibility strong model	Vantage sensitivity strong model	Vantage sensitivity weak model	Differential susceptibility weak model
Intercept	59.94***	57.58***	58.87***	56.43***
Cross-over point (*C*)	23.64***	27 (fixed)	27 (fixed)	23.25***
Prenatally exposed to depression	0 (fixed)	0 (fixed)	0.23	−0.17
Not prenatally exposed to either SSRIs or depression	1.09***	0.74***	0.88***	1.09***
Not prenatally exposed to depression but SSRI-exposed	2.07***	1.39***	1.54***	2.10***
*R*^2^	0.22	0.20	0.21	0.22
AIC (smaller is better)	592.94	593.79	595.11	594.74

SSRI, selective serotonin reuptake inhibitor; AIC, Akaike information criteria.

a. *n* = 139 reflects that measures of postnatal depression were not used in this model since it had been shown in a previous model not to contribute to self-regulation at 6 years.

b. In the vantage sensitivity strong model, *F*(2,136) = 17.41***; in the differential susceptibility strong model, *F*(3,135) = 12.69***; in the vantage sensitivity weak model, *F*(3,135) = 11.80***; and in the differential susceptibility weak model, *F*(4,134) = 9.51***.

***P < 0.001.

For the differential susceptibility strong model, the intercept was significant (β = 56.94, s.e. = 1.12, *P* < 0.0001). In particular, the slope for children of non-depressed, non-SSRI-treated mothers was significant (β = 1.09, s.e. = 0.27, *P* < 0.0001), the slope for children of non-depressed SSRI-treated mothers was significant (β = 2.07, s.e. = 0.57, *P* = 0.0004) and the cross-over point was significant (β = 23.64, s.e. = 1.44, *P* < 0.0001, 95% CI 20.79–26.49), with a range of possible values (CHAOS score is between 15 and 27). *R*^2^ for the model was 0.22. In contrast, the slope for children of depressed mothers was 0.

For the vantage sensitivity strong model, the intercept was significant (β = 57.58, s.e. = 1.05, *P* < 0.0001). The slope for children of depressed mothers was 0, whereas the slope for those of non-depressed and non-SSRI-exposed mothers was significant (β = 0.74, s.e. = 0.16, *P* < 0.0001), the slope for children of non-depressed and SSRI-exposed mothers was significant (β = 1.38, s.e. = 0.28, *P* < 0.0001) and the cross-over point was fixed at the maximum CHAOS score.^16^ The *R*^2^ for the model was 0.20.

## Discussion

At 6 years of age children of mothers who experienced symptoms of mild to severe depression during pregnancy had generally poorer executive functions than children of mothers who were not symptomatic during pregnancy (Fig. 1). Moreover, executive functions remained stable, even in the context of higher home chaos and regardless of whether mothers had been treated with an SSRI during pregnancy. In contrast, children of mothers who showed fewer depressive symptoms during the third trimester were affected by home chaos, but the degree to which they were affected depended on prenatal exposure to an SSRI. At lower levels of household chaos, SSRI-exposed children had better executive function skills compared with non-exposed children, but as chaos increased, executive functions suffered among SSRI-exposed children. Thus, both prenatal depressed maternal mood and SSRI exposure appeared to shape different responses to home environment. However, it is also conceivable that home environment may have been more chaotic because of child dysregulation and in this sense, chaos could have also been a predictor of child executive function, and the relationship between chaos and child development may in fact be bidirectional.

### Susceptibility to household chaos

The magnitude of the cross-over point (i.e. the point where prenatal depressed mood was affected by level of household chaos) between patterns of executive functions and increasing levels of household chaos at 6 years in our competitive-confirmatory models suggested the possibility of differential susceptibility^15^ (Fig. 2). Namely, to the left of the cross-over point, at lower levels of chaos, children of mothers who were not particularly symptomatic during their pregnancies had better executive functions than children whose mothers were symptomatic during pregnancy. To the right of the cross-over point, at higher levels of chaos, the converse was true: children of less depressed mothers had worse executive functions than children of depressed mothers. Additionally, children of mothers who were treated with an SSRI but were not depressed during pregnancy (as measured with a HRSD score < 8) and perhaps derived some therapeutic benefit from pharmacotherapy, were more susceptible to household chaos than children of mothers who were not prenatally depressed and not SSRI-treated.

Children of prenatally depressed mothers (regardless of SSRI exposure) were not susceptible (solid green line) to household chaos. However, children of non-depressed mothers living with a lower level of household chaos differed in their response to a calmer environment. Namely, children of non-depressed mothers showed better executive functions than children of prenatally depressed mothers, but only where Confusion, Hubbub and Order Scale (CHAOS) scores were <23.6 (left-side vertical bar on horizontal line). In contrast, children of non-depressed mothers where CHAOS scores were >26.5 had significantly higher Behavior Rating Inventory of Executive Function (BRIEF) scores (poorer executive functions compared with children of depressed mothers (right-side vertical bar on horizontal line)).

Prenatal exposure to SSRI antidepressants to treat mood disturbances during pregnancy have added another developmental risk.^6^ Given that serotonergic networks play a prominent role in the development of the prefrontal cortex, our findings might also reflect early changes in serotonin signalling. Converging evidence from animal models and human research links increased 5-HT signalling with improved cognition.^21^ In animal models, increased intrasynaptic 5-HT, secondary to genetic variations, may improve cognitive functions and cognitive flexibility.^22^ In humans, similar genetic variations have been associated with improved attentional inhibition, cognitive flexibility, memory and reaction time.^8^

### Maternal mood during early childhood

Our findings also highlight the importance of assessing maternal mood long after delivery and its impact on child executive functions.^2,23^ Previous studies report that greater maternal depressive symptoms when the child is aged 2 were associated with poorer performance on executive function tasks in children at age 3, and poorer executive functions performance at age 3 predicted increased externalising symptoms at age 6.^24^ Higher levels of maternal depression in early childhood are associated with poorer executive functions in offspring at age 18 months,^22^ which appear to extend to poorer attentional control at 8 years.^25^

Parental scaffolding, or support of development of new skills, is viewed as critical to the development of children's executive functions,^26^ suggesting that goal-directed behaviour could be disrupted in chaotic home environments. Home environments that are more chaotic have been associated with fewer and lower-quality caregiver interactions.^23^ Greater environmental confusion in the home environment was associated with lower executive function scores in 4-year-olds.^27^ A number of conceptual frameworks have been proposed to explain how environment predicts development. Until recently, attention has focused on a diathesis–stress framework, whereby some individuals are more vulnerable to negative events because of an inherent ‘vulnerability’ (e.g. psychological trait or ‘risk gene’).^28^ The diathesis–stress model asserts that some individuals carry ‘risk’ factors (e.g. depression, genotype) that make them disproportionately susceptible to adverse environments.^28^ The model implies that, in the absence of adversity, there should be no differences between vulnerable and resilient individuals, whereby some individuals are disproportionately adversely affected by negative exposures, while others may conceivably benefit from supportive and enriching experiences.

### The vantage sensitivity and differential susceptibility models

To date, studies have focused on searching for ‘main effects’ outcomes related to prenatal SSRI exposure. This has led to conflicting findings that offer limited insight into why some, but not all, children are at risk, or who might even benefit from maternal antidepressant treatment during gestation. To account for the negative effects of contextual adversity, the differential susceptibility model has been proposed to account for susceptible individuals who are not just vulnerable to adversity, but are more developmentally plastic or malleable.^29^ The differential susceptibility model asserts that some individuals are susceptible to both the ‘dark side’ as well as the ‘bright side' of environmental exposures.^8,15,29^ However, for others a vantage sensitivity may make them disproportionately susceptible to positive environments and they may actually benefit from positive or supportive factors (i.e. genetic variations) or environments.^28^ In the current study, children of mothers who were not depressed during pregnancy were susceptible to both lower and higher levels of home chaos. Under less chaotic environments, they had better executive functions than children of depressed mothers in a similar setting, suggesting a ‘vantage sensitivity’. In a positive environment, susceptible children with such a vantage sensitivity could do very well, but as adversity increased, these children's behaviour suffered. For some children, maternal prenatal depression may be a ‘buffer’ against a subsequently chaotic home environment, whereas for others (i.e. with prenatal SSRI exposure), susceptibility to the home environment is increased. This may reflect a positive impact of maternal antidepressant treatment, which might confer a long-term benefit to the child under some, but not all, circumstances.^15^

### Limitations

Key limitations need to be mentioned. We could not control the sample size available for this follow-up component of our prospective longitudinal cohort study. The available sample may have constrained the power needed to test two- and three-way interactions, and larger studies will need to confirm our findings. Moreover, the use of maternal report of child executive functions raises the possibility that depressed mood may have biased behavioural observations; however, consistency between teacher and parent report has been previously reported.^18^ The study lacked information about the effect of fathers, maternal partners or significant others in shaping household chaos. Our use of dichotomised maternal mood measures could misclassify mood status and may not reflect the impact of patterns of mood trajectories across pre- and postnatal periods, which have been examined by Park et al.^30^

Further, SSRI use in pregnancy is inherently related to maternal depression, but not all women in our cohort appeared to benefit from prenatal SSRI treatment. Some women in the SSRI group were relatively asymptomatic during pregnancy (i.e. HRSD scores <8) and this might reflect that SSRI treatment resulted in symptom remittance. However, variations in maternal illness severity still existed and unmeasured maternal characteristics may have made them inherently different from those who were treated with an SSRI and remained symptomatic. In particular, CHAOS scores for SSRI-treated women who had relatively low levels of depressive symptoms (prenatal HRSD < 8 score, *n* = 14), compared with SSRI-treated women who remained symptomatically depressed (HRSD > 8; *n* = 33), were not substantially different. Although an HRSD score >8 reflected the presence of mild to moderate depressive symptoms, we were not able to determine associations between her mood disturbance and everyday function. Moreover, this symptom range (i.e. >8) maybe too broad, as it included both mild and severe symptoms and could therefore be subject to many confounders, such as treatment adherence, drug dosing and comorbid maternal conditions. Further, in this study we examined the association of SSRI treatment (maternal) in pregnancy on child behaviour (executive functions) 6 years later, which likely reflects the impact of prenatal SSRI exposure on the developing fetal brain. Conceivably, this is a different context from exposure to an SSRI in a mature brain, suggesting that executive functions may be affected in a different way than when the exposure occurs during periods of early brain development. Finally, distinguishing the impact of maternal mood (pre- and postnatal) from SSRI exposure is challenging in cohort studies where exposures cannot be randomised nor can the impact of variations in the severity of maternal mood be controlled for introducing possible ‘confounding by indication’.

### Implications

This study was undertaken to further examine variations in early child behaviour in the context of maternal mental illness, to understand how exposure to maternal depressed mood (pre- and postnatal) and the antidepressants used to treat these disorders may shape child executive functions and whether these exposures are associated with a susceptibility to household chaos in 6-year-olds. Maternal depressed mood and antidepressant treatment during pregnancy have been associated with a child's emerging ability to adapt to environmental demands and engage in goal-directed behaviour, known as executive functions. Why some, but not all, exposed children are affected remains a key question. Although depressed maternal mood prenatally was associated with lower executive functions in offspring at 6 years of age, the degree of susceptibility to household environment appeared to depend both on whether the mothers were prenatally depressed and whether they had been treated with an SSRI. Children exposed to SSRIs prenatally were more susceptible, but the impact depended on levels of home chaos. Although prenatal depressed maternal mood may ‘buffer’ a child against a chaotic home environment, prenatal SSRI exposure did not appear to confer a protection against a susceptibility to household confusion. Namely, children not exposed to prenatal maternal depressed mood had better executive functions at lower levels of chaos, but when chaos increased, demonstrated poorer executive functions. Interventions to test whether better treatment of maternal depression during pregnancy and in the early postnatal period, as well as modifying household chaos, improve developmental outcomes in this setting are warranted.

## Data Availability

The data that support the findings of this study are available on request from the corresponding author, T.F.O. The data are not publicly available due to privacy/ethical restrictions.
